# Electromechanical factors associated with favourable outcome in cardiac resynchronization therapy

**DOI:** 10.1093/europace/euac157

**Published:** 2022-09-15

**Authors:** Francesco Maffessanti, Tomasz Jadczyk, Jacek Wilczek, Giulio Conte, Maria Luce Caputo, Krzysztof S Gołba, Jolanta Biernat, Magdalena Cybulska, Guido Caluori, François Regoli, Rolf Krause, Wojciech Wojakowski, Frits W Prinzen, Angelo Auricchio

**Affiliations:** Center for Computational Medicine in Cardiology, Università della Svizzera Italiana, Lugano, Switzerland; Maria Cecilia Hospital, GVM Care and Research, Cotignola, Italy; Division of Cardiology and Structural Heart Diseases, Medical University of Silesia, Katowice, Poland; Interventional Cardiac Electrophysiology Group, International Clinical Research Center, St. Anne’s University Hospital Brno, Brno, Czech Republic; Department of Electrocardiology and Heart Failure, Medical University of Silesia, Katowice, Poland; Division of Cardiology, Istituto Cardiocentro Ticino, Lugano, Switzerland; Division of Cardiology, Istituto Cardiocentro Ticino, Lugano, Switzerland; Department of Electrocardiology and Heart Failure, Medical University of Silesia, Katowice, Poland; Department of Electrocardiology and Heart Failure, Medical University of Silesia, Katowice, Poland; Department of Electrocardiology and Heart Failure, Medical University of Silesia, Katowice, Poland; IHU LIRYC, Electrophysiology and Heart Modeling Institute, Fondation Bordeaux Université, University of Bordeaux & INSERM, U 1045,Cardiothoracic Research Center of Bordeaux, Pessac, France; Division of Cardiology, Istituto Cardiocentro Ticino, Lugano, Switzerland; Cardiology Service, Ospedale Regionale di Bellinzona e Valli, Bellinzona, Switzerland; Center for Computational Medicine in Cardiology, Università della Svizzera Italiana, Lugano, Switzerland; Euler institute, Università della Svizzera Italiana, Lugano, Switzerland; Division of Cardiology and Structural Heart Diseases, Medical University of Silesia, Katowice, Poland; Department of Physiology, CARIM, Maastricht University, Maastricht, The Netherlands; Center for Computational Medicine in Cardiology, Università della Svizzera Italiana, Lugano, Switzerland; Division of Cardiology, Istituto Cardiocentro Ticino, Lugano, Switzerland

**Keywords:** Cardiac resynchronization therapy, Electromechanics, Left-bundle branch block, Activation time, Interventricular delay, Intraventricular conduction

## Abstract

**Aims:**

Electromechanical coupling in patients receiving cardiac resynchronization therapy (CRT) is not fully understood. Our aim was to determine the best combination of electrical and mechanical substrates associated with effective CRT.

**Methods and results:**

Sixty-two patients were prospectively enrolled from two centres. Patients underwent 12-lead electrocardiogram (ECG), cardiovascular magnetic resonance (CMR), echocardiography, and anatomo-electromechanical mapping (AEMM). Remodelling was measured as the end-systolic volume (ΔESV) decrease at 6 months. CRT was defined effective with ΔESV ≤ −15%. QRS duration (QRSd) was measured from ECG. Area strain was obtained from AEMM and used to derive systolic stretch index (SSI) and total left-ventricular mechanical time. Total left-ventricular activation time (TLVAT) and transeptal time (TST) were derived from AEMM and ECG. Scar was measured from CMR. Significant correlations were observed between ΔESV and TST [rho = 0.42; responder: 50 (20–58) vs. non-responder: 33 (8–44) ms], TLVAT [−0.68; 81 (73–97) vs. 112 (96–127) ms], scar [−0.27; 0.0 (0.0–1.2) vs. 8.7 (0.0–19.1)%], and SSI [0.41; 10.7 (7.1–16.8) vs. 4.2 (2.9–5.5)], but not QRSd [−0.13; 155 (140–176) vs. 167 (155–177) ms]. TLVAT and SSI were highly accurate in identifying CRT response [area under the curve (AUC) > 0.80], followed by scar (AUC > 0.70). Total left-ventricular activation time (odds ratio = 0.91), scar (0.94), and SSI (1.29) were independent factors associated with effective CRT. Subjects with SSI >7.9% and TLVAT <91 ms all responded to CRT with a median ΔESV ≈ −50%, while low SSI and prolonged TLVAT were more common in non-responders (ΔESV ≈ −5%).

**Conclusion:**

Electromechanical measurements are better associated with CRT response than conventional ECG variables. The absence of scar combined with high SSI and low TLVAT ensures effectiveness of CRT.

## Graphical Abstract

**Graphical Abstract:**
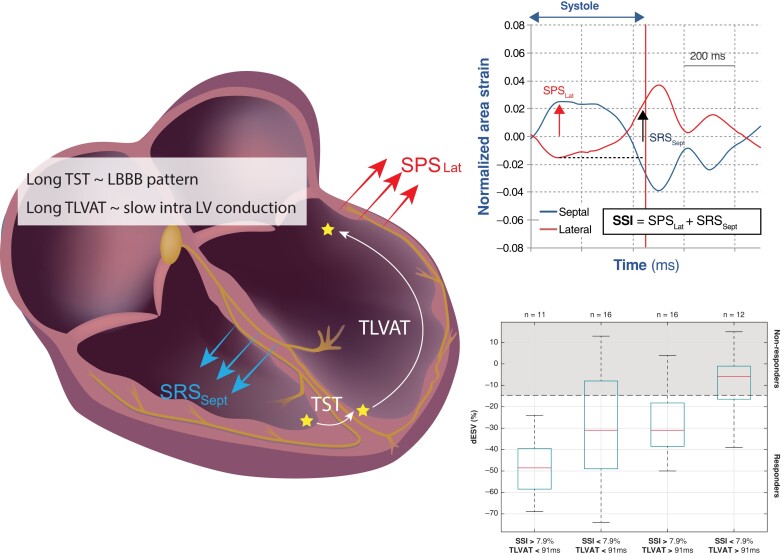

**What’s new?**

- A long total left-ventricular activation time (TLVAT) indicates slow myocardial conduction within the LV and negatively affects reverse remodelling after CRT.
- A long transeptal time reflects the presence of left-bundle branch block and is associated with reverse remodelling.
- A high systolic stretch index (SSI), an index of mechanical dyssynchrony, constitutes a favourable mechanical substrate for CRT.
- TLVAT, SSI, and scar burden are independent factors contributing to reverse remodelling.

## Introduction

Over the last two decades randomized controlled trials showed that cardiac resynchronization therapy (CRT) creates a consistent benefit in selected patients with heart failure (HF).^1^ Nowadays, CRT can treat 25–30% of symptomatic HF patients (NYHA Classes II–IV) and it is recommended in the individuals presenting with electrical conduction delay characterized by QRS duration (QRSd) ≥ 130 ms and, most preferably, left-bundle branch block (LBBB). Despite these stringent selection criteria, the benefit of CRT varies considerably among the patients.^2^

Recent studies indicate that mechanical discoordination parameters (reciprocal shortening and stretching) provide valuable information on top of the conventional measures of electrical dyssynchrony, to identify the substrate for CRT.^3^ In daily clinical practice, the CRT candidate is routinely assessed using different modalities, such as electrocardiogram (ECG), echocardiography, and cardiac magnetic resonance (CMR).^4^ However, combining the information obtained from these diagnostic tools is problematic due to inaccuracy in matching of the data over space and time.^5^

A technique that provides full electromechanical (EM) mapping is the NOGA-XP system (Biologic Delivery Systems, Division of Biosense Webster, a Johnson & Johnson Company, Irwindale, CA, USA). Beside regular electrograms along the endocardium, the measurement of the motion of the tip of the electrode at all positions provides *in vivo* quantification of local mechanical deformations at exactly the same position and at the same time.^6^

It was the aim of the present study to use the NOGA-XP system to find the best combination of electrical and mechanical parameters associated with the echocardiographic response to CRT. To this purpose, we designed a two-centre, 6-month follow-up study, aiming at: (i) comparing the baseline EM characteristics between CRT responders and non-responders; (ii) evaluating the strength of the association between EM variables and the extent of reverse remodelling; and (iii) identifying the independent EM predictors of positive CRT outcome.

## Methods

### Study population

Sixty-two patients with moderate to severe HF were prospectively enrolled in the study. All patients were referred for implantation of a CRT device at two centres, Istituto Cardiocentro Ticino (*n* = 29, Lugano, Switzerland) and the Medical University of Silesia (*n* = 33, Katowice, Poland) between 2012 and 2018.

Inclusion criteria were: age >18 years; ischaemic, and non-ischaemic cardiomyopathy; sinus rhythm; QRSd > 120 ms; QRS morphology of LBBB (criteria by Strauss *et al*.)^7^; and/or intraventricular conduction delay. Exclusion criteria were: presence of coronary artery disease requiring revascularization; acute coronary syndrome <3 months prior to study enrolment; implantation of pacemaker; presence of implantable cardioverter-defibrillator; left-ventricular thrombus or aneurysm; severe aortic stenosis, renal failure (glomerular filtration rate <30 mL/min/1.73 m^2^); history of neoplasm; bleeding diathesis; HIV, hepatitis B virus, hepatitis C virus infection; pregnancy; and contrast allergy.

The study was approved by the Institutional Review Board of each participating centre, and written informed consents were obtained by each patient before the enrolment. Patient information was de-identified and data were transferred in accordance with General Data Protection Regulation regulations.

### Study design

At the time of enrolment, patients were in optimal pharmacological therapy, NYHA Class II/III, and left-ventricular ejection fraction (LVEF) <35%.

At baseline, each patient underwent standard 12-lead echocardiography, CMR examination, standard 2D echocardiography and NOGA-based invasive EM study prior to CRT implantation. Left-ventricular volumes and function were reassessed 6 months after the implantation by means of standard 2D echocardiography to evaluate clinical outcome of CRT.

### Electromechanical mapping

Electromechanical mapping of the left endocardial cavity was performed using the NOGA-XP system (Biologic Delivery Systems, Division of Biosense Webster a Johnson & Johnson Company) and a conventional 7 Fr deflectable-tip mapping catheter (NAVI-STAR, Biosense Webster). The mapping and navigation systems have been described in detail elsewhere.^4,6^ The catheter recorded unipolar and bipolar electrograms at 1 kHz, as well as the position of the contact point at 100 Hz. Care was taken to cover the whole endocardial cavity. The acquired electrical and mechanical signals at different positions were aligned over time by the system with respect to the simultaneously recorded 12-lead surface ECG.

### Definitions and post processing of electromechanical data

**Figure 1:**
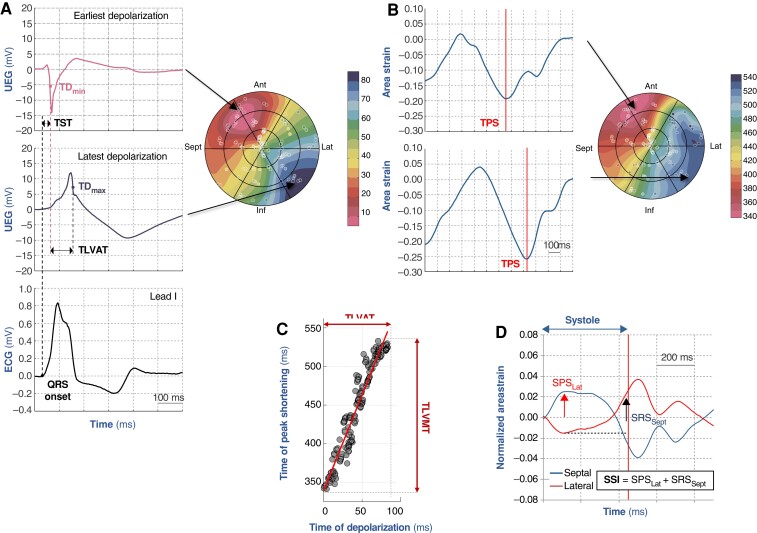
Schematic of electromechanical measurements. (*A*) Identification of local time of depolarization (TD) and its left-ventricular endocardial distribution in a bullseye view. Transeptal time (TST) is defined as the time interval between the onset of the QRS complex and the earliest TD measured in the left-ventricular endocardial cavity. Total left-ventricular time (TLVAT) is defined as the time interval between the earliest and latest TD. (*B*) Identification of the local time-to-peak shortening (TPS) as the earliest peak of the local area strain curve. (*C*) The time intervals between the earliest and latest TD or TPS define TVLAT and the total left-ventricular activation mechanical time (TLVMT). The regression line between TD and TPS describes the electromechanical coupling. (*D*) Schematic for the computation of the systolic stretch index (SSI). After normalizing the strain curves by subtracting the average strain curve, SSI is defined as the sum of the early systolic posterolateral stretch (SPS_Lat_) and the late septal systolic rebound stretch (SRS_Sept_). See text for details.

The acquired data sets were subsequently imported and processed in MATLAB (Mathworks, USA) to obtain a set of EM measurements to be investigated as factors associated with the procedural outcome. Data were filtered removing all those points showing non-physiological motion (excessive spatial excursion, open trajectories, and/or catheter sliding) or belonging to the papillary muscles and protruding inside the reconstructed cavity. For the electrical activity, the local time of depolarization (TD) was identified in correspondence of the steepest change of the local unipolar electrogram in a time window belonging to the QRS complex (*Figure 1A*). For the mechanical contraction, local area strain was computed following a procedure previously described.^4^ The first strain peak was used to identify the time-to-peak shortening (TPS) interval (*Figure 1B*). From the processed signals a set of EM measurements were derived:

1. EM coupling, defined as the Pearson’s correlation coefficient *R* between local TD and TPS^6^ (*Figure 1C*);
2. transeptal time (TST), a measure of interventricular delay, defined as the time interval between the onset of the QRS complex and the earliest LV endocardial depolarization (*Figure 1A*);
3. total left-ventricular activation time (TLVAT), a measure of intra-LV conduction delay, determined as the time interval between the earliest and latest LV endocardial depolarization (TD_max_ − TD_min_; *Figure 1A* and *C*);
4. TST/TLVAT ratio was considered to describe the proportion between interventricular and intraventricular conduction intervals;
5. total mechanical activation time (TLVMT), a measure of mechanical dyssynchrony, determined as the time interval between earliest and latest negative peak of area strain (TPS_max_ − TPS_min_; *Figure 1C*);
6. systolic stretch index (SSI), a measure of mechanical discoordination, defined as the sum of the posterolateral pre-stretch and the septal rebound stretch (*Figure 1D*) of the area strain curve during the ejection phase.^8,9^

### Cardiovascular magnetic resonance

Cardiovascular magnetic resonance was performed with a 3 T scanner (MAGNETOM Skyra; Siemens Healthcare, Germany) at Cardiocentro Ticino and a 1.5 T scanner at Medical University of Silesia (SIGNA; GE Medical Systems, USA), equipped with standard torso coil. A stack of delayed enhancement short-axis images covering the whole long axis were obtained 7–12 min after the intravenous bolus injection of gadolinium (0.2 mmol/kg body weight). The epicardial and endocardial boundaries were manually traced in each slice and then scar was automatically identified using the full-width half-maximum criterion.^10^ The scar burden was expressed as the per cent volume of scar with respect to the total myocardial volume.

### Transthoracic echocardiography

Echocardiographic examinations were performed using iE33 (Philips Medical Systems, Andover, MA, USA; Cardiocentro Ticino) and Epiq 7G (Philips Ultrasound, Inc, Reedsville, PA, USA; Medical University of Silesia) ultrasound systems. Patients were evaluated in the left lateral decubitus position and images acquired from standard parasternal, suprasternal, and apical windows. Data were digitally recorded and analysed offline by a single observer for the two centres. Left-ventricular end-systolic and end-diastolic volumes, and ejection fraction were obtained using the modified biplane Simpson method.

### Endpoint

Left-ventricular (LV) remodelling at 6-month follow up was measured in terms of per cent change in end-systolic volume with respect to the baseline value (ΔESV). Significant response to CRT was defined as a reduction in ESV ≥15% at 6-month evaluation.

### Statistical analysis

Data are expressed as median (first to third quartiles), or as absolute count (and per cent frequency), as appropriate. Comparison between groups has been evaluated by means of Mann–Whitney *U* test or Fisher’s exact test for continuous and categorical variables, respectively. The strength of the association between EM variables and the extent of remodelling at 6-month follow up (ΔESV) was quantified by Spearman’s correlation coefficient (rho). Area under the curve (AUC) was determined from receiver operating characteristic (ROC) analysis to determine the ability of the baseline EM variables to classify the response to CRT. The association between single factor and CRT response was also evaluated using binary logistic regression and expressed in terms of odds ratio. Factors significantly associated with CRT response at univariate analysis were entered in a stepwise multivariate binary logistic regression. A *P*-value of <0.05 was considered statistically significant. Statistical analysis was performed using SPSS (IBM, Armonk, NY, USA).

## Results

Out of the 62 enrolled patients, 4 patients did not undergo CRT, 2 were excluded for poor acoustic window either at baseline or at follow-up echocardiographic evaluation and one patient did not show up at follow up. The baseline clinical characteristics of the remaining 55 patients, overall as well as separately for the two centres, are summarized in *Table 1*. The patients in the two centres were similar in terms of clinical characteristics, except for a larger prevalence of ischaemic cardiomyopathy and LBBB in Centre #2.

**Table 1:** Patient characteristics

	Overall	Centre #1	Centre #2	*P*-value
*Demographics*				
Number of patients	55	22	33	
Male	42 (76)	16 (73)	26 (79)	0.60
Ischaemic cardiomyopathy	43 (78)	10 (45)	33 (100)	**<0**.**01**
NYHA (II/III)	27(49)/28(51)	10(45)/12(55)	17(52)/16(48)	0.66
*ECG characteristics*				
Sinus rhythm	45 (82)	19 (86)	26 (79)	0.48
Atrial fibrillation	10 (18)	3 (14)	7 (21)	0.48
Heart rate (b.p.m.)	70 (60–76)	72 (64–83)	67 (60–75)	0.15
QRS duration (ms)	158 (143–176)	162 (139–180)	158 (144–170)	0.56
LBBB	51 (93)	18 (82)	33 (100)	**0**.**01**
IVCD	4 (7)	4 (18)	0 (0)	**0**.**03**
*CMR LV function*				
EF (%)	25 (21–32)	25 (20–32)	26 (21–32)	0.77
Mass (g)	157 (136–184)	156 (140–175)	160 (131–192)	0.96
EDV (mL)	248 (214–314)	250 (178–320)	243 (224–310)	0.68
ESV (mL)	191 (147–224)	182 (141–240)	194 (157–219)	0.65
*Medications*				
ACE inhibitors	49 (89)	21 (95)	28 (85)	0.22
Diuretics	48 (87)	18 (82)	30 (91)	0.32
Beta-blockers	55 (100)	22 (100)	33 (100)	—

Data expressed as median (1st–3rd) quartiles for continuous variables or count (%) for categorical variables. *P*-value refers to Centre #1 vs. Centre #2, Mann–Whitney *U* test or Fisher’s exact test when appropriate. Bold values refers to significant p-values (*p* < 0.05), consistently with the description in the statistical analysis sub-section.

CMR, cardiovascular magnetic resonance; ECG, electrocardiogram; EDV, end-diastolic volume; EF, ejection fraction; ESV, end-systolic volume; IVCD, interventricular conduction disturbance; LBBB, left-bundle branch block; NYHA, New York Heart Association.

Endocardial mapping (approximately 30 min/patient) and CRT implantation were feasible in all patients with no peri- and post-procedural complications. For each patient, 225 (179–251) unique LV endocardial sites were mapped and used for post processing. Mean fluoroscopy and radiation dose were 22 min 26 s and 322.8 mGy, respectively.

### Echocardiographic response to cardiac resynchronization therapy

Cardiac resynchronization therapy resulted in a significant reduction of EDV [from 230 (180–274) to 196 (145–250) mL] and ESV [from 170 (131–205) to 112 (81–170) mL], and an increase in LVEF [from 25 (23–30)% to 39 (32–46) %]. With respect to the endpoint, the observed reduction in ΔESV was 34 (7–47) mL, with 39 patients (71%) experiencing a reduction in ΔESV(%) ≥15%, and thus classified as responders.

### Baseline electromechanical characteristics in cardiac resynchronization therapy responders and non-responders

*Table 2* shows that at baseline responders had lower scar burden, longer TST, shorter TLVAT, and higher SSI values than non-responders. Conversely, QRSd, LVEF, and TLVMT where similar between the two groups.

**Table 2:** Comparison between electrical and mechanical variables in the responder and non-responder subgroups

	Responder (*n* = 39)	Non-responder (*n* = 16)	*P*-value
EF (%)	26 (23–31)	23 (22–28)	0.70
EM coupling (unitless)	0.80 (0.63–0.88)	0.83 (0.60–0.89)	0.94
QRS duration (ms)	155 (140–176)	167 (155–177)	0.11
TST (ms)	50 (20–58)	33 (8–44)	**0**.**04**
TLVAT (ms)	81 (73–97)	112 (96–127)	**<0**.**01**
TST/TLVAT (%)	55 (23–84)	29 (6–43)	**0**.**01**
TLVMT (ms)	123 (95–163)	145 (115–161)	0.28
SSI (unitless)	10.7 (7.1–16.8)	4.2 (2.9–5.5)	**<0**.**01**
Scar (%)	0.0 (0.0–1.2)	8.7 (0.0–19.1)	**0**.**01**

Data expressed as median (1st–3rd) quartiles, or expected value (95% confidence interval). Bold values refers to significant p-values (*p* < 0.05), consistently with the description in the statistical analysis sub-section.

*P*-value refers to responder vs. non-responder, Mann–Whitney *U* test.

CI, confidence interval; EF, ejection fraction; EM, electromechanical coupling: Pearson’s *R* of TD-TPS; TLVAT, total left-ventricular activation time; TLVMT, total left-ventricular mechanical time; TD, time of depolarization; TPS, time-to-peak shortening; TST, transseptal time.

### Relation between extent of left-ventricular remodelling and baseline electromechanics

Significant correlations were observed between %ΔESV and TST, TLVAT, scar burden, and SSI (*Table 3*), while LVEF, EM coupling, QRSd, and TLVMT did not significantly relate to reverse remodelling. Importantly, higher TST and SSI values were associated with a larger reduction in ESV, whereas larger scar burden and more prolonged TLVAT related to a lower extent of remodelling.

**Table 3:** Correlation between left-ventricular reverse remodelling (ΔESV) and baseline electromechanical variables

	Spearman’s rho (*P*-value)	AUC (95% CI)
EF	0.16 (0.25)	0.59 (0.44–0.74)
QRS duration	−0.13 (0.35)	0.64 (0.48–0.78)
TST	0.42 (<0.01)	0.68 (0.51–0.81)
TLVAT	−0.68 (<0.01)	0.87 (0.75–0.95)
TST/TLVAT	0.56 (<0.01)	0.74 (0.58–0.86)
TLVMT	−0.08 (0.56)	0.60 (0.45–0.73)
SSI	0.41 (<0.01)	0.81 (0.68–0.91)
EM coupling	−0.09 (0.54)	0.51 (0.33–0.68)
Scar	−0.27 (0.03)	0.71 (0.56–0.87)

Receiver operating characteristic analysis for electromechanical measurements in identifying significant response to CRT (ΔESV > 15%). Positive rho values indicate a larger reduction in ESV with increasing value of the related variable.

AUC, area under the curve; CI, confidence interval; EF, ejection fraction; EM coupling: Pearson’s *R* of TD-TPS; SSI, systolic stretch index; TLVAT, total left-ventricular activation time; TLVMT, total left-ventricular mechanical time; TD, time of depolarization; TPS, time-to-peak shortening; TST, transseptal time.

The performance of EM measurements in identifying responders is shown in *Table 3*. The best discriminatory capability was found for TLVAT and SSI (AUC > 0.80). Scar burden and TST moderately predicted the response to CRT (AUC 0.71 and 0.68, respectively).

### Independent electromechanical predictors of cardiac resynchronization therapy response

**Figure 2:**
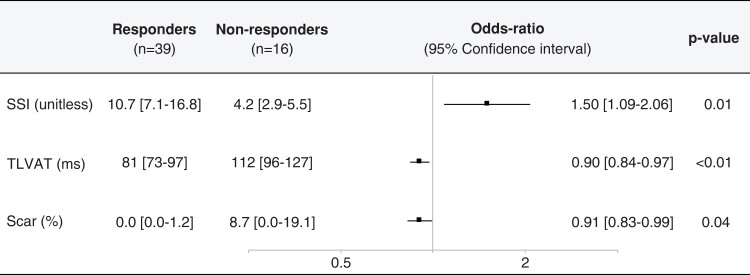
Multivariate binary logistic regression of factors associated with 6-month positive response to cardiac resynchronization therapy.

Multivariate logistic regression analysis identified TLVAT, scar burden, and SSI as independent factors associated with significant response to CRT (*Figure 2*). Of note, regression analysis performed on the subgroup of patients with any scar showed a poor, although significant, correlation between TLVAT and the per cent scar burden (*R*^2^ = 0.20, *P* = 0.04), suggesting that the slow intraventricular conduction can only be marginally explained by the presence of scar.

**Figure 3:**
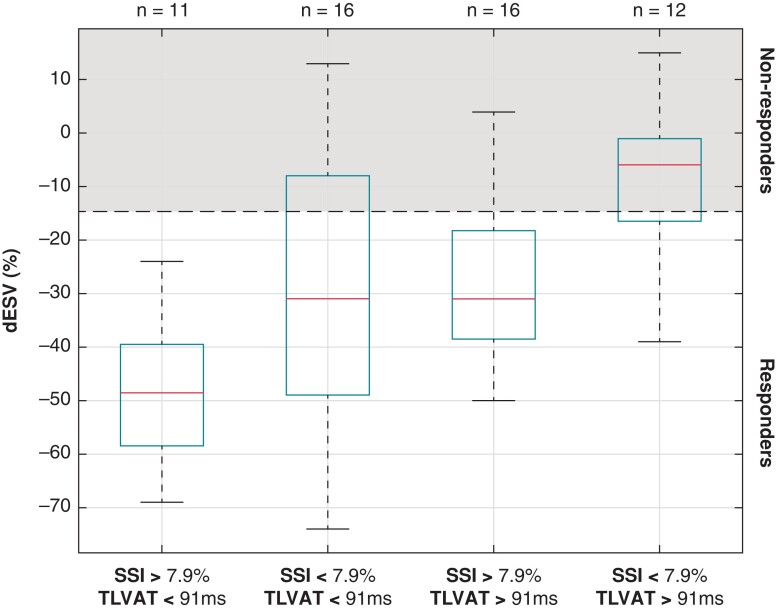
Overview of in left-ventricular end-systolic volume change (ΔESV) in patients grouped according to the systolic stretch index (SSI) and total left-ventricular activation time (TLVAT) with respect to the median values observed in the study population. The central mark in the box indicates the median, and the bottom and top edges of the box indicate the 25th and 75th percentiles, respectively. The whiskers extend to the most extreme data points not considered outliers.

Focusing on the EM variables TLVAT and SSI, patients were subdivided into subgroups according to the median values of these variables in the study population (*Figure 3*). Subjects with high SSI and short TLVAT all responded to CRT with a median reduction in LVESV of ∼50%. Conversely, most of the subjects with prolonged TLVAT and low SSI fell in the non-response area (median reduction in LVESV ∼5%). Intermediate reductions on LVESV were observed in the subgroups with low SSI and TLAVT and with high SSI and TLVAT.

## Discussion

The main findings of the present study are that: (i) of the electrical measures, TLVAT and TST have opposite relationships with reverse remodelling, (ii) a large SSI value constitutes a favourable mechanical substrate for CRT; (iii) TLVAT, SSI, and scar burden are independent factors contributing to reverse remodelling.

### Electrical substrate of cardiac resynchronization therapy response

A novel finding of the present study is that a long TLVAT seems to hamper reverse remodelling. This seems at odds with the idea that longer QRSd enhances CRT response. However, more recently, LBBB morphology rather than QRSd was found to be predictive of CRT response.^11^ The latter also supports the other main finding of the present study, that TST relates to reverse remodelling, because a long TST, with RV activation preceding LV activation, is a specific feature of LBBB.

In contrast, TLVAT describes the time required for the electrical impulse to travel along the entire LV endocardium. The mean TLVAT values of 81 and 112 ms in responders and non-responders, respectively, are considerably larger than can be expected to be caused by conduction along the Purkinje fibres. Therefore, TLVAT in these patients probably reflects the impulse conduction through the LV working myocardium. Prolongation of this impulse conduction may be caused by fibrosis or other kinds of electrical uncoupling. These poor conduction properties persist during biventricular pacing, therefore a long baseline TLVAT likely translates into a slower conduction during pacing, hampering CRT effectiveness. These findings appear in conflict with the positive correlation between reverse remodelling and LV activation time, calculated as QRSd minus RV activation time (time until the first notch in the QRS complex of the LBBB morphology on a surface ECG).^12^ Therefore, these discrepant findings are likely due to the difference in definitions and therefore we deliberately called this variable TLVAT, expressing that the mapping covered the entire LV endocardium.

More recent mapping studies have shown that in two-thirds of LBBB patients, the location of the block is proximal in the Purkinje system, because pacing in the His-bundle or left-bundle branch can significantly shorten QRSd.^13^ We propose that a long TST reflects LBBB and a short TLVAT good LV conduction properties and that also enhance relatively fast activation during biventricular pacing. Therefore, patients with a high TST/TLVAT ratio are most likely patients with a proximal LBBB.

### Mechanical substrate of cardiac resynchronization therapy response

The superiority of SSI over other mechanical markers (TPS, TLVMT) to predict CRT response is in agreement with those reported by previous studies.^8,14^ This good performance can be explained considering the two components of SSI, early systolic LV lateral wall stretch and mid-systolic septal rebound stretch: both are dependent septal-to-lateral wall contraction dyssynchrony, likely induced by an LBBB activation pattern, as well as preserved myocardial contractility.^8^ The latter likely takes into account the presence of scar, which is known to be associated with poor response, both in patients with ischaemic and non-ischaemic cardiomyopathy.^15^

### Interplay between electromechanical substrates

While we hypothesized that the combination of electrical and mechanical measures would improve the association with CRT response, this turned out not to be true for the relation between time of depolarization and peak shorting (TD–TPS relation). In a previous study, Maffessanti *et al*.^16^ showed that presence of scar impairs EM coupling, even when scar is remote from late activated segments. Furthermore, Jadczyk *et al*.^17^ presented data confirming influence of EM coupling on kinetics and rotational pattern in patients with HF and LBBB. The poor performance of the TD–TPS relation in predicting CRT response may be explained by the limitations of TPS as mechanical marker, because in particular in the septum its measurement is complicated by multiple peaks.^14^ While poor TD–TPS relations in other studies might have been caused by difficulties in spatial or temporal alignment of the different measurements, in the present study all measurements were performed exactly at the same time and location. These unique *in vivo* data sets provided results that could be used as a reference for other studies based on conventional mapping systems and imaging techniques, such as strain ultrasound.

On the other hand, combining electrical (TLVAT) and mechanical (SSI) variables improves the prediction of CRT response. While the excellent response in patients with small TLVAT and large SSI and the poor response in those with large TLVAT and small SSI were expected, it was worth noticing that reverse remodelling was comparable in the two intermediate categories, suggesting the need of a combined EM evaluation in the selection of CRT patients. Wouters *et al.*^18^ showed a similar additive predictive value for septal rebound stretch (the major component of SSI) and QRSarea, the area under the QRS complex in vectorcardiograms.

### Potential implications

Current measurements were performed using invasive measurements, but TLVAT and SSI may be assessed with less invasively. The recently introduced ultra-high frequency ECG provides a measure of conduction velocity underneath the precordial electrodes, called Vd, which may be indicative of TLVAT.^19^

Speckle tracking echocardiography is already well established. However, reliable SSI determination depends on proper timing of end systole, because especially septal strain may change considerably around this time (see *Figure 1*). Some investigators used single (septal and lateral) wall imaging at a smaller angel to achieve a higher sampling rate.^14^

Albeit the present study focused on CRT, the characterization of the EM substrate could help identifying the patients most amenable to conduction system pacing (CSP), which is emerging as a valid method for delivering effective ventricular resynchronization.^13^ Accordingly, the NOGA-XP mapping system allows for spatio-temporal assessment of both electrical and mechanical activation patterns. This information may help to stratify patients referred to resynchronization therapy based on EM characteristics, i.e. (i) preserved/dispersed EM coupling and/or (ii) concordant/discordant latest electrical and mechanical activation regions. Furthermore, based on NOGA study results and coronary venous anatomy, a feasibility of optimal lead placement and pacing strategy could be simulated pre-procedurally supporting personalized clinical approach on conventional CRT vs. CSP.

### Limitations

Study population of this study is relatively small compared with randomized trials on CRT. However, the novelty of the study relies on the approach adopted to derive EM measures based on mapping data, without the need of multimodality integration. Also, although scar burden was assessed from CMR, the current gold standard, this did not require spatial integration and could eventually be derived from mapping.^4^ The study focused on the effects of EM substrate only, and did not consider other procedural correlates, such as lead positioning. Furthermore, we used a traditional definition of CRT response as ΔESV(%) ≥15%. Other studies employed other echocardiographic parameters (i.e. LV end-diastolic diameter),^20^ but ΔESV is the most commonly used index in large clinical trials corresponding with mortality and HF-related hospitalizations.^21^

## Conclusion

Effectiveness of CRT significantly relies on the EM substrate and the scar burden. A combined EM approach was able to identify, despite similar ECG morphology, the opposite effects of TLVAT and TST on the reverse remodelling, the first associated with interventricular delay, the latter pointing to an adverse effect of slow intraventricular conduction on the left side.

## Data Availability

The data underlying this article cannot be shared publicly due to the privacy reasons of individuals that participated in the study. The data will be shared on reasonable request to the corresponding author.

## References

[1] Prinzen FW , Vernooy K, Auricchio A. Cardiac resynchronization therapy: state-of-the-art of current applications, guidelines, ongoing trials, and areas of controversy. Circulation 2013;128:2407–18. 24276876 10.1161/CIRCULATIONAHA.112.000112

[2] McDonagh TA , Metra M, Adamo M, Gardner RS, Baumbach A, Böhm M et al 2021 ESC guidelines for the diagnosis and treatment of acute and chronic heart failure. Eur Heart J 2021;42:3599–726. 34447992 10.1093/eurheartj/ehab368

[3] Wouters PC , Leenders GE, Cramer MJ, Meine M, Prinzen FW, Doevendans PA et al Acute recoordination rather than functional hemodynamic improvement determines reverse remodelling by cardiac resynchronisation therapy. Int J Cardiovasc Imaging 2021;37:1903–11. 33547623 10.1007/s10554-021-02174-7 PMC8255256

[4] Maffessanti F , Prinzen FW, Conte G, Regoli F, Caputo ML, Suerder D et al Integrated assessment of left ventricular electrical activation and myocardial strain mapping in heart failure patients: a holistic diagnostic approach for endocardial cardiac resynchronization therapy, ablation of ventricular tachycardia, and biological therapy. JACC Clin Electrophysiol 2018;4:138–46. 29600778 10.1016/j.jacep.2017.08.011

[5] Pavo N , Jakab A, Emmert MY, Strebinger G, Wolint P, Zimmermann M et al Comparison of NOGA endocardial mapping and cardiac magnetic resonance imaging for determining infarct size and infarct transmurality for intramyocardial injection therapy using experimental data. PLoS One 2014;9:e113245. 25409528 10.1371/journal.pone.0113245 PMC4237404

[6] Kroon W , Lumens J, Potse M, Suerder D, Klersy C, Regoli F et al In vivo electromechanical assessment of heart failure patients with prolonged QRS duration. Heart Rhythm 2015;12:1259–67. 25748674 10.1016/j.hrthm.2015.03.006

[7] Strauss DG , Selvester RH, Wagner GS. Defining left bundle branch block in the era of cardiac resynchronization therapy. Am J Cardiol 2011;107:927–34. 21376930 10.1016/j.amjcard.2010.11.010

[8] Lumens J , Tayal B, Walmsley J, Delgado-Montero A, Huntjens PR, Schwartzman D et al Differentiating electromechanical from non-electrical substrates of mechanical discoordination to identify responders to cardiac resynchronization therapy. Circ Cardiovasc Imaging 2015;8:e003744. 26338877 10.1161/CIRCIMAGING.115.003744

[9] Gorcsan J III , Anderson CP, Tayal B, Sugahara M, Walmsley J, Starling RC et al Systolic stretch characterizes the electromechanical substrate responsive to cardiac resynchronization therapy. JACC Cardiovasc Imaging 2019;12:1741–52. 30219394 10.1016/j.jcmg.2018.07.013

[10] Flett AS , Hasleton J, Cook C, Hausenloy D, Quarta G, Ariti C et al Evaluation of techniques for the quantification of myocardial scar of differing etiology using cardiac magnetic resonance. JACC Cardiovasc Imaging 2011;4:150–6. 21329899 10.1016/j.jcmg.2010.11.015

[11] Moss AJ , Hall WJ, Cannom DS, Klein H, Brown MW, Daubert JP et al Cardiac-resynchronization therapy for the prevention of heart-failure events. N Engl J Med 2009;361:1329–38. 19723701 10.1056/NEJMoa0906431

[12] Sweeney MO , van Bommel RJ, Schalij MJ, Borleffs CJ, Hellkamp AS, Bax JJ. Analysis of ventricular activation using surface electrocardiography to predict left ventricular reverse volumetric remodeling during cardiac resynchronization therapy. Circulation 2010;121:626–34. 20100970 10.1161/CIRCULATIONAHA.109.894774

[13] Upadhyay GA , Cherian T, Shatz DY, Beaser AD, Aziz Z, Ozcan C et al Intracardiac delineation of septal conduction in left bundle-branch block patterns. Circulation 2019;139:1876–88. 30704273 10.1161/CIRCULATIONAHA.118.038648

[14] Lumens J , Leenders GE, Cramer MJ, De Boeck BW, Doevendans PA, Prinzen FW et al Mechanistic evaluation of echocardiographic dyssynchrony indices: patient data combined with multiscale computer simulations. Circ Cardiovasc Imaging 2012;5:491–9. 22661491 10.1161/CIRCIMAGING.112.973446

[15] Díez J , González A, Kovacic JC. Myocardial interstitial fibrosis in nonischemic heart disease, part 3/4: JACC focus seminar. J Am Coll Cardiol 2020;75:2204–18. 32354386 10.1016/j.jacc.2020.03.019 PMC7213023

[16] Maffessanti F , Jadczyk T, Kurzelowski R, Regoli F, Caputo ML, Conte G et al The influence of scar on the spatio-temporal relationship between electrical and mechanical activation in heart failure patients. Europace 2020;22:777–86. 31942982 10.1093/europace/euz346

[17] Jadczyk T , Kurzelowski R, Golba KS, Wilczek J, Caluori G, Maffessanti F et al Local electromechanical alterations determine the left ventricle rotational dynamics in CRT-eligible heart failure patients. Sci Rep 2021;11:3267. 33547401 10.1038/s41598-021-82793-1 PMC7865069

[18] Wouters PC , van Everdingen WM, Vernooy K, Geelhoed B, Allaart CP, Rienstra M et al Does mechanical dyssynchrony in addition to QRS area ensure sustained response tocardiac resynchronization therapy? Eur Heart J Cardiovasc Imaging 2021. https://academic.oup.com/ehjcimaging/advance-article/doi/10.1093/ehjci/jeab264/6454061?login=true. Online ahead of print. 10.1093/ehjci/jeab264 PMC9671288 34871385

[19] Jurak P , Curila K, Leinveber P, Prinzen FW, Viscor I, Plesinger F et al Novel ultra-high-frequency electrocardiogram tool for the description of the ventricular depolarization pattern before and during cardiac resynchronization. J Cardiovasc Electrophysiol 2020;31:300–7. 31788894 10.1111/jce.14299

[20] Guo Z , Liu X, Cheng X, Liu C, Li P, He Y et al Combination of left ventricular End-diastolic diameter and QRS duration strongly predicts good response to and prognosis of cardiac resynchronization therapy. Cardiol Res Pract 2020;2020:1257578. 32411441 10.1155/2020/1257578 PMC7201746

[21] Chung ES , Gold MR, Abraham WT, Young JB, Linde C, Anderson C et al The importance of early evaluation after cardiac resynchronization therapy to redefine response: pooled individual patient analysis from 5 prospective studies. Heart Rhythm 2022;19:595–603. 34843964 10.1016/j.hrthm.2021.11.030

